# Poly(levodopa)-Modified β-(1 → 3)-D-Glucan Hydrogel Enriched with Triangle-Shaped Nanoparticles as a Biosafe Matrix with Enhanced Antibacterial Potential

**DOI:** 10.3390/molecules31010181

**Published:** 2026-01-03

**Authors:** Anna Michalicha, Vladyslav Vivcharenko, Anna Tomaszewska, Magdalena Kulpa-Greszta, Barbara Budzyńska, Dominika Fila, Judit Buxadera-Palomero, Agnieszka Krawczyńska, Cristina Canal, Dorota Kołodyńska, Anna Belcarz-Romaniuk, Robert Pązik

**Affiliations:** 1 Chair and Department of Biochemistry and Biotechnology, Medical University of Lublin, Chodzki 1, 20-093 Lublin, Poland; anna.belcarz-romaniuk@umlub.edu.pl; 2 Department of Tissue Engineering and Regenerative Medicine, Medical University of Lublin, Chodzki 1, 20-093 Lublin, Poland; vladyslav.vivcharenko@umlub.edu.pl; 3 Faculty of Biotechnology, Collegium Medicum, University of Rzeszow, Pigonia 1, 35-310 Rzeszow, Poland; atomaszewska@ur.edu.pl (A.T.); mkulpa@ur.edu.pl (M.K.-G.); 4 Independent Laboratory of Behavioral Studies, Medical University of Lublin, Chodzki 1, 20-093 Lublin, Poland; barbara.budzynska@umlub.edu.pl; 5 Department of Inorganic Chemistry, Institute of Chemical Sciences, Faculty of Chemistry, Maria Curie-Skłodowska University, Maria Curie-Skłodowska Sq. 2, 20-031 Lublin, Poland; dominika.fila@mail.umcs.pl (D.F.); dorota.kolodynska@mail.umcs.pl (D.K.); 6 Biomaterials, Biomechanics and Tissue Engineering Group, Department of Materials Science and Engineering and Institute for Research and Innovation in Health (IRIS), Universitat Politècnica de Catalunya-BarcelonaTech (UPC), Av. Eduard Maristany 10–14, 08019 Barcelona, Spain; judit.buxadera@upc.edu (J.B.-P.); cristina.canal@upc.edu (C.C.); 7 Barcelona Research Center in Multiscale Science and Engineering (CCEM), Universitat Politècnica de Catalunya-BarcelonaTech (UPC), 08034 Barcelona, Spain; 8 Faculty of Materials Science and Engineering, Warsaw University of Technology, 02-507 Warsaw, Poland; agnieszka.krawczynska@pw.edu.pl; 9 Centro de Investigación Biomédica en Red de Bioingeniería, Biomateriales y Nanomedicina (CIBER-BBN), Instituto de Salud Carlos III, 28029 Madrid, Spain

**Keywords:** antimicrobial biomaterials, poly(L-DOPA), wound dressings, shape-defined nanoparticles, heat generation effects

## Abstract

Biomaterials derived from natural-origin polymers often lack the desired functionality and additional features, such as antibacterial properties, which could be beneficial in the design of modern wound dressings. Our research aimed to fabricate biosafe antibacterial dressings through the modification of curdlan-based hydrogels with triangle-shaped silver nanoparticles (AgTNPs) and poly(L-DOPA) (PL). The prepared hydrogels, including physicochemical, structural, biological, and antibacterial assessments, were thoroughly characterized. All formulations were confirmed to be non-toxic toward eukaryotic cells. The presence of PL in the hydrogels significantly reduced mortality in the zebrafish larvae model, highlighting the improved biocompatibility of the hydrogels. The three-component hydrogel (CUR-PL-AgT) demonstrated a high antibacterial effectiveness against *Staphylococcus aureus* and *Pseudomonas aeruginosa*. Additionally, the same three-component material outperformed a hydrogel containing only AgTNPs in promoting blood clot formation. Furthermore, PL enhanced the heat generating capability of hydrogels, showing their potential in medical applications where the temperature effects can stimulate biological processes of different natures.

## 1. Introduction

Curdlan is a linear β-(1 → 3)-D-glucan that, after hydration and heating at 80–100 °C, forms a high-set thermal nonreversible hydrogel of significant strength and elasticity for different purposes. Recently, curdlan has attracted growing attention for its biomedical and pharmaceutical applications. There are two main strategies for producing curdlan-based derivatives with improved biomedical properties. One of them applies different chemical modification methods of multiple hydroxyl groups in repeating glucose units of the curdlan backbone. These techniques, including carboxymethylation, sulfonation, phosphorylation, etc. [1], allow for the fabrication of water-soluble derivatives with specific biological properties. For example, cholesterol-conjugated carboxymethyl curdlan was used to produce nanoparticles for epirubicin delivery [2]; aminated β-1,3-glucan stimulated in vivo wound healing in diabetes [3], curdlan sulfates exhibited significant anti-HIV activity [4,5], and partially hydrolyzed curdlan showed strong antitumor activity [6]. However, all of these modifications lead to the formation of curdlan derivatives with increased solubility, which results in the loss of the stable hydrogel structure; thus, these derivatives are useless for producing biomaterials, e.g., hydrogels for wound dressings. The second strategy in the design of curdlan-based derivatives involves synthesizing an intact curdlan hydrogel (by gel-casting, thermal gelation, or freeze-drying) and adding other important functional compounds. These approaches make it possible to obtain stable hydrogels suitable for biomaterial preparation, but are less effective at enhancing curdlan’s bioactivity. However, in some cases, curdlan gel modified in this way retains its water-insolubility, mechanical properties, and acceptable functionality. Examples are a hybrid chitosan–β-1,3-glucan bone scaffold matrix for enhanced osteoblast adhesion, spread, and proliferation [7], and an elastic hydroxyapatite–curdlan filler for bone tissue regeneration [8,9,10].

Silver nanoparticles (AgNPs) are frequently proposed as functional compounds to enhance the utility of hydrogels for medical applications. This strategy offers several important advantages, such as increased antimicrobial efficacy, enhanced wound healing attributes, efficient drug delivery mechanisms, and increased mechanical strength. AgNPs are well-known for their straightforward synthesis, adaptable shapes, large surface area, and proficiency in topical drug administration and applications related to wound healing [11,12].

Among AgNPs, those with a triangular shape offer distinct advantages over other morphologies, such as increased surface area [13,14]. This enables more efficient surface contact between nanoparticles and bacterial cells. Consequently, this attachment enhances the permeability of the bacterial membrane, which ultimately leads to its disintegration [11,15,16]. Furthermore, the defined shape allows for precise control over nanoparticle interactions within the hydrogel matrix, potentially improving structural integrity and performance [17]. Other than that, it is necessary to remember that AgNPs are also very interesting due to their outstanding plasmonic properties, which can be fine-tuned by manipulating particle size and morphology. This tunability opens up a broad range of possibilities for AgNP application in sensing, photonics, and modern therapeutic approaches, including photothermal treatments [18,19]. Of particular importance for the biological applications involving optical stimulation is the wavelength of the irradiating light source. It should be within a so-called optical biological window, preferably the NIR range, due to its low photon energy, deeper tissue penetration, and minimal absorption or scattering by biological tissue [20]. AgNPs can be fabricated in such a way that their optical absorption will cover that range. However, it is practically impossible to achieve that with spherical Ag morphologies, thus a triangular shape for the metallic silver particles is necessary to achieve the shift of absorbance toward NIR [18]. At the same time, Ag particles can benefit from dual functionality: an optical response that enables the photothermal heating of the system, and significant antibacterial activity. It has been demonstrated that spherical AgNPs are especially attractive for antibacterial applications; nevertheless, triangular silver nanostructures also show considerable antibacterial activity [21,22,23]. Therefore, incorporating triangular AgNPs into a hydrogel matrix could be beneficial for targeted purposes, as is the case for curdlan hydrogel.

Previously, we have shown that the modification of curdlan hydrogels with poly(L-DOPA) (PL) is crucial for efficient antibiotic bonding and the resulting antibacterial activity of the hydrogel [24]. Thus, we hypothesized that modifying the curdlan hydrogel matrix with shape-defined silver nanoparticles (AgTNPs) and PL could enhance its biomedical applicability. This study aims to investigate the potential synergistic effects of PL and AgTNPs on the properties of a curdlan hydrogel, strongly emphasizing the hydrogel’s biomedical utility, including biocompatibility, antibacterial properties, heat generation ability, and surface properties.

## 2. Results and Discussion

### 2.1. Characterization of the Silver Nanoparticles

**Figure 1:**
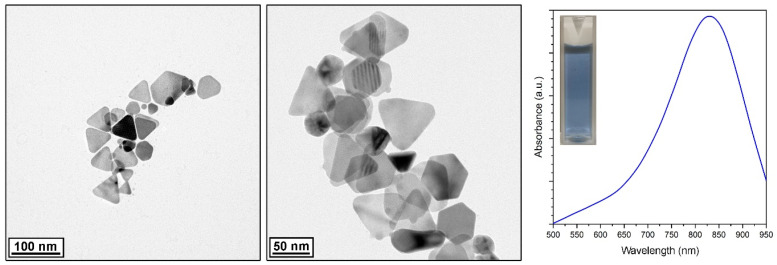
TEM images (**left**) and absorption spectra (**right**) of the prism-shaped silver nanoparticles.

The size and shape of the synthesized silver nanoparticles were examined by TEM imaging (Figure 1). As can be noticed, most of the AgNPs present a triangular (prism-shaped) morphology with an average particle size of 53.8 ± 7.9 (nm). The optical properties of the nanoparticles were evaluated by measuring the absorption spectrum of the colloidal suspension, as shown in Figure 1 (right). A strong and broad absorption peak within the 550–950 nm spectral range was observed, with a maximum located at around 825 nm, being characteristic of prism morphology as reported earlier [25]. The location of the absorption band for this type of metallic particle fits perfectly with the so-called first biological optical window (700–980 nm). Therefore, AgTNPs can be beneficially used in biological applications since NIR stimulation will be particle-specific due to the significantly reduced absorption of the biological system [20]. It is well-known that the absorption of metallic NPs is directly related to strong localized surface plasmon resonance (LSPR). One of the consequences of LSPR relies on the heating ability of AgNPs upon energy dissipation, which can be a basis for photothermal and photodynamic therapies (PTT and PDT) or drug release applications [26].

### 2.2. Heat Generation and Active Substance Release Process

AgTNPs show effective light-to-heat conversion due to the strong surface plasmon resonance effect followed by biologically preferential NIR light absorption. Among many other types of materials with the same light-induced ability to produce significant heating effects, some organic molecules have also been successfully applied [20,26,27]. For instance, the L-DOPA molecule has an intense absorption band within the NIR range, making it a potentially interesting candidate for applications in which temperature effects are essential. Several authors have already shown that L-DOPA, except for its biological activities, can generate the biologically relevant temperatures necessary for the stimulation of the cell response [28,29,30,31,32,33]. Thus, AgNPs and L-DOPA are extremely promising agents for temperature-stimulated biological processes. AgNPs are known for their multifunctional properties and can show strong antibacterial activity as well as acting as a SERS probe (surface-enhanced Raman scattering) for the detection of analytes [21,34]. Similarly, L-DOPA is a precursor of dopamine and holds significant potential as a dopamine replacement agent, for instance, in Parkinson’s disease treatment [35,36,37,38].

**Figure 2:**
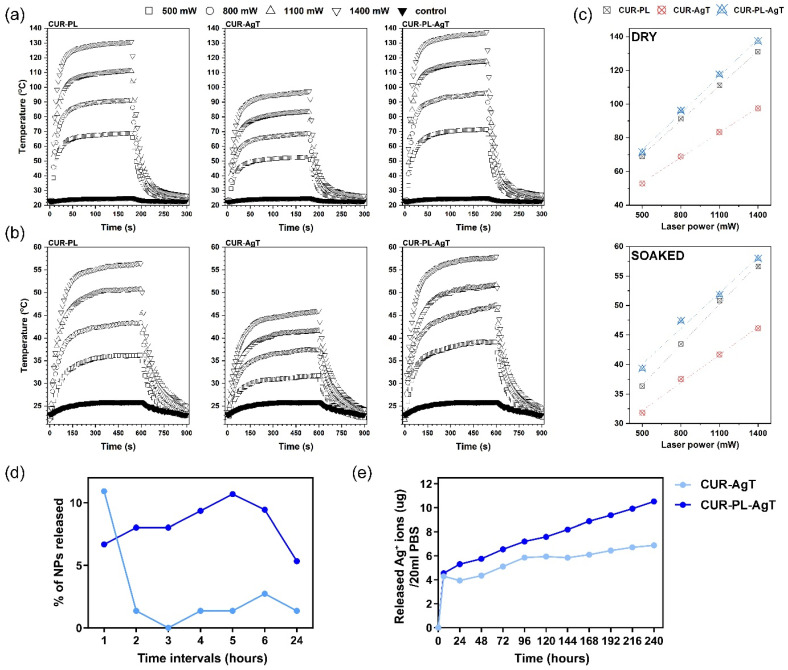
Heat induction of the dry (**a**) and soaked (**b**) nanocomposites under the action of the 808 nm NIR laser, as well as the laser power dependence of the dry and soaked hydrogels (**c**). Pure curdlan hydrogel (dry or soaked, respectively) was used as a control sample. Nanoparticle release as % of total load (**d**), and Ag^+^ ion release from curdlan hydrogels (**e**).

Characterizations of laser light conversion into thermal energy within the first biological optical window were carried out on binary (CUR-PL and CUR-AgT) and ternary (CUR-PL-AgT) hydrogels. For all samples tested, the measurements were conducted on dry and wet materials (Figure 2a,b). Composites soaked with Ringer’s solution mimic the natural tissue environment at the implantation site or in the wound bed (after the absorption of wound exudates). Heat generation (Figure 2a,b) was carried out as a function of laser power in the range of 500–1400 mW with a laser optical density (LOD) within 1000–2800 mW/cm^2^. Dry hydrogels have shown efficient conversion even at low laser power regimes. The maximum temperature obtained exceeded 43 °C at a 500 mW stimulation. Upon comparison of the binary composites, the CUR-PL sample showed better heat generation ability than the CUR-AgT-containing hybrid. This may be due to the difference in the amount of optically active materials, the intensities of the absorption abilities of both compounds, and the scattering effect on AgNPs. However, both samples were allowed to reach relevant temperatures. It has to be noted that *T_max_* was achieved rapidly and could minimize the thermotolerance effect in biological applications. One can observe (in Figure 2a,b) that the ternary composite has shown comparable heat generation capacity to CUR-PL hydrogels. This means that the energy conversion is exclusively affected by the optical behavior of the PL. Some slight differences in *T_max_* are more likely to be associated with the repeatability of the measurements, temperature sensing errors, or laser light stability. Therefore, for the ternary hydrogels, the presence of the AgTNPs can be therapeutically justified in terms of possible synergy with other functionalities, i.e., antibacterial activity. All samples showed a linear temperature dependence with laser power, with *T_max_* values of 131, 97, and 137 °C for CUR-PL, CUR-AgT, and CUR-PL-AgT dry hydrogels, respectively. Control over laser power can be used to optimize the heating effect.

Experiments on Ringer’s solution-soaked hybrids were performed since the porous hydrogels will absorb tissue fluids after implantation or exposure to wound exudates. As was expected, due to the presence of a water-based medium within the hydrogel microstructure, the conversion of light into heat decreased the *T_max_* in all cases. This is because these hybrids contain significant water molecules with a high specific heat capacity (4.185 J/g°C). However, the achieved temperature for the wet samples is still within an acceptable range in terms of inducing biological effects at 43 °C at a low laser power (800 mW), at least for the CUR-PL and CUR-PL-AgT composites (Figure 2b). The lowest energy-to-heat conversion was shown by a binary composite with AgTNPs that needed higher laser power to reach 43 °C. A further temperature increase in the CUR-AgT sample is possible through increased Ag particle concentration. This solution might also strengthen the antibacterial effect in a final application. It was also shown that *T_max_* showed clear linear dependence upon laser stimulation of either dry or wet composites, while the saturation limit was not achieved (Figure 2c). The consequent laser power increase will lead to a higher *T_max_*. Of course, one has to consider safe exposure limits and the overall stability of the curdlan to avoid its degradation.

Controlled release of Ag^+^ and AgTNPs from hydrogel matrices can significantly enhance the antibacterial effectiveness of the designed hydrogel matrices [39,40]. Furthermore, it has previously been proven that the shape and size of silver nanoparticles matter regarding the antibacterial properties of the modified matrices [41]. Therefore, it was justified to evaluate the release of both AgTNPs (Figure 2d) and silver ions (Ag^+^) (Figure 2e) from the designed matrices. A rapid release of approximately 11% of silver nanoparticles (Figure 2d) from the CUR-AgT hydrogel microstructure occurred within the first hour of the experiment. Then, the concentration of AgNPs in the medium dropped significantly. This may suggest the fast dissolution, aggregation, or precipitation of the released NPs. In the case of the PL-modified sample, the release was more stable and lasted up to 5 h, reaching approx. 11% of the total content; then the concentration of NPs decreased to approx. 5%. This phenomenon may be caused by PL protecting and stabilizing the nanoparticles incorporated into the hydrogel structure, ensuring their prolonged release. The cumulative profiles of Ag^+^ release (Figure 2e) from PL-modified (CUR-PL-AgT) and unmodified (CUR-AgT) hydrogels showed that silver ions were gradually released into the PBS medium for at least 240 h. A higher amount of released Ag^+^ ions was noted for the PL-modified hydrogel variant.

### 2.3. Mechanical and Surface Properties

The mechanical properties of hydrogels can be crucial and must be considered in matrix engineering for pharmaceutical and biomedical applications [42]. Hydrogels with sufficient strength are significantly protected against mechanical damage and external forces, minimizing the risk of further tissue injury, particularly for wound dressings. Additionally, their structural stability ensures that the dressing remains intact in a moist wound environment, while their flexibility facilitates adaptation to body movements without compromising durability [43]. As expected, the mechanical profiles of the reference matrices (CUR and CUR-PL) remained consistent with our previous report [32], confirming the reproducibility of the material and measurement conditions.

**Figure 3:**
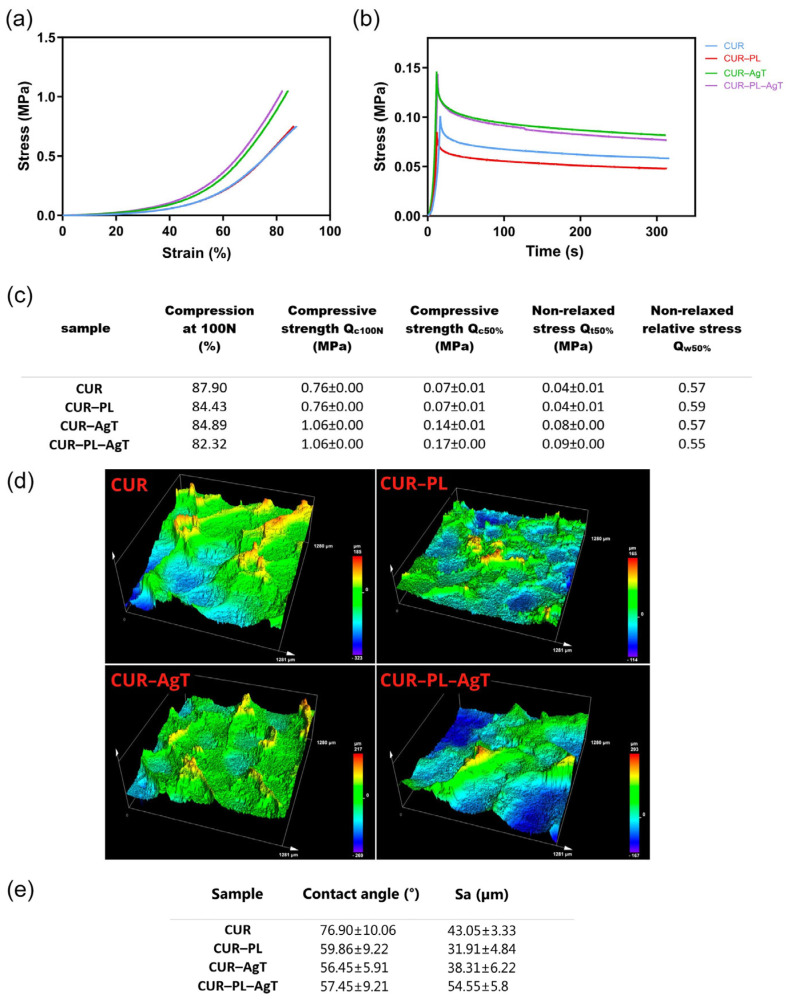
Stress–strain curves (**a**), relaxation curves (**b**), and mechanical parameters (**c**) of hydrogel samples (*σ_c_*_100N_—compressive strength measured as the maximum stress at 100 N, *σ_c_*_50%_—compressive strength as the maximum stress at 50 N, *σ_t_*_50%_—non-relaxed stress, and *σ_w_*_50%_—non-relaxed relative stress calculated as the ratio *σ_t_*_50%_/*σ_c_*_50%_). (**d**) Biomaterial surface topographies obtained by laser scanning microscopy analysis. (**e**) The contact angle measurements and surface area roughness (Sa) of the tested materials.

In the study by Jiang et al. [44], the incorporation of AgNPs into a hydrogel matrix led to threefold and ninefold increases in tensile and compressive strength, respectively. Therefore, the curdlan hydrogels were tested to verify the effect of AgTNPs on their mechanical properties. Both the control hydrogel (CUR) and the PL-modified hydrogel (CUR-PL) showed similar stress–strain and relaxation curve profiles (Figure 3a,b), indicating that poly(L-DOPA) modification alone did not significantly affect the mechanical response of the curdlan matrix.

However, hydrogel modification with AgTNPs significantly improved mechanical properties, manifesting as consistent mechanical strength curves. This effect agrees with the available literature [45]. The three-component hydrogel (CUR-PL-AgT) exhibited an approximately 5% lower compression at 100 N compared to the control curdlan sample (CUR), indicating reduced deformability under a fixed compressive load (Figure 3c).

It is worth noting that compressive strength (defined as the maximum stress at a 50% compression—*σ*_*c*50%_) was twice as high for samples modified with AgTNPs than without them. These results demonstrate the beneficial effect of silver nanoparticles on the mechanical properties of matrices. The investigated hydrogels are soft and can undergo substantial deformation without the loss of mechanical integrity. The stress relaxation observed under constant strain indicates that the materials gradually release applied stress, which is advantageous for wound dressing applications where gentle contact with soft tissues and tolerance to mechanical loading are required.

The hydrophilic surface of a potential wound dressing is essential for its functionality. It impacts the biocompatibility of the material [46] and assures suitable liquid (wound exudate) absorption properties [47]. Biopolymers such as polydopamine are known to form an adhesive layer with hydrophilic properties. Thus, evaluation of the effect of poly(L-DOPA) addition on the surface properties is crucial [48]. In this study, all tested samples revealed satisfactory hydrophilic characteristics with a contact angle lower than 90°. It was shown that all hydrogel modifications reduced that parameter and increased hydrophilicity (Figure 3e).

Images of biomaterials’ surface topography are presented in Figure 3d, whereas the average surface roughness is shown in Figure 3e. The blue areas indicate the deepest valleys observed on material surfaces, while red represents surface peaks. The CUR-PL biomaterial was characterized by the lowest surface roughness of 31.91 ± 4.84 µm. The most desirable roughness was noted for the CUR-PL-AgT hydrogel (54.55 ± 5.8 µm).

### 2.4. Characterization of the Porosity

The porosity of the hydrogel plays an essential role in its properties, especially in the context of controlled release of active substances. The increase in porosity of the hydrogel leads to an increased surface area in contact with the surrounding medium, which can accelerate the release of active substances [49]. For example, Podhorská et al. developed a double-porosity hydrogel modified with RGDS oligopeptide (Arg-Gly-Asp-Ser), significantly improving cell adhesion and proliferation, which is crucial for tissue engineering [50]. It was also proven that the high porosity of gelatin hydrogels not only enhanced their swelling ability, allowing for greater fluid absorption, but also facilitated a more efficient controlled drug release [51].

**Figure 4:**
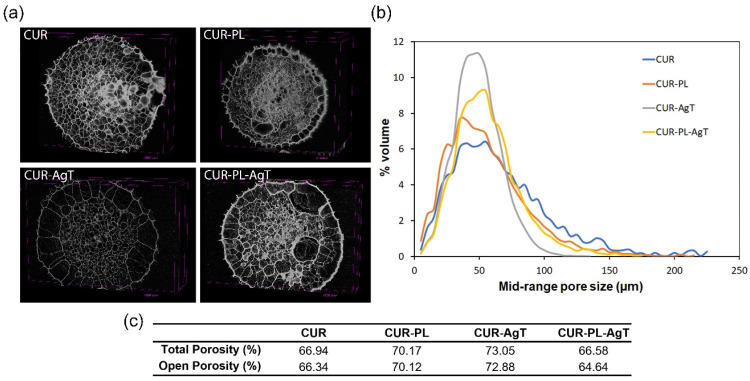
(**a**) Images obtained by micro-CT of a transversal section, (**b**) pore size distribution, and (**c**) total porosity and open porosity of the samples as determined from the whole matrices by micro-CT.

The pore size distribution of the manufactured curdlan hydrogel matrices showed a maximum around 50 µm for all samples (Figure 4b), while the maximum pore size was observed in the 135 to 225 µm range. The narrowest distribution was found for the CUR-AgT and the CUR-PL-AgT samples, while the CUR and CUR-PL showed a wider distribution of pore size (Table S1, complemented by Figure 4b). In general, all samples show a total porosity within the same range. Interestingly, most of the pores were interconnected, as shown by the fact that the open porosity of all samples is practically equal to the total porosity (Figure 4c). Therefore, nanoparticle incorporation did not significantly alter the porosity characteristics of the curdlan matrices, as additionally reflected by the extracted image of the hydrogels’ transversal sections (Figure 4a).

### 2.5. Cytotoxicity and Toxicity in Fibroblasts and Zebrafish Model

**Figure 5:**
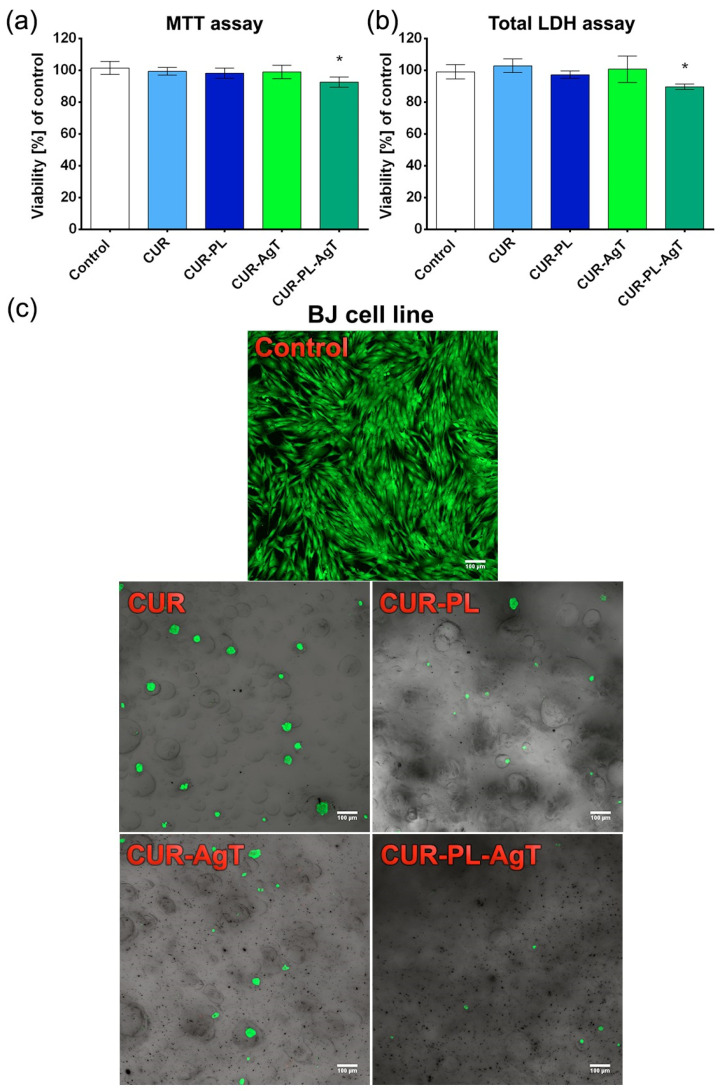
Biocompatibility evaluation of the biomaterials: (**a**) MTT cytotoxicity test conducted according to ISO 10993-5; (**b**) total LDH test conducted according to ISO 10993-5 (* statistically significant results compared to the polypropylene control, *p* value < 0.05, one-way ANOVA followed by Tukey’s test); (**c**) live/dead staining of BJ cell line cultured on the produced materials for 48 h (green fluorescence represents viable fibroblasts, while red fluorescence represents dead cells).

Biocompatibility is the most important feature of wound dressing, as a cytotoxic environment caused by an applied material can potentially impair and exacerbate the healing process [52]. Understanding the biological safety through cell line experiments for potential wound dressings is essential for predicting their behavior in a biological environment and minimizing risks to patient health. Previous studies on the modification of a curdlan hydrogel with poly(L-DOPA) using the Before Gelling (BG) method showed that the PL-modified hydrogel was non-toxic toward skin fibroblast cells [24]. Considering these results, the safety of AgTNP addition to the poly(L-DOPA)-modified curdlan structure was tested in vitro using human skin fibroblasts (BJ), indirectly by the MTT and LDH total assays, and through a direct-contact test (live/dead fluorescent staining). The MTT assay conducted after 24 h of the treatment of fibroblasts with the extracts of the tested materials revealed that all samples were non-toxic. All materials were characterized by high cell viability, exceeding 89%. The CUR-PL-AgT sample showed a statistically significant reduction in cell viability of 85% compared to the control (Figure 5a). Nevertheless, according to ISO 10993-5:2009, a material is considered cytotoxic only when cell viability decreases below 70%. 

High sample biocompatibility was also observed in the LDH total assay. The lowest cell biomass was noted for sample CUR-PL-AgT with cell viability at 89% compared to the control (Figure 5b). A lack of cytotoxicity was observed in the direct-contact live/dead test. After 2 days of culturing normal human skin fibroblasts (BJ cells) on the tested biomaterials, only a few viable green-stained cells were observed under confocal laser scanning microscopy (Figure 5c), with no red-stained dead cells detected using propidium iodide. The live/dead test confirmed that BJ fibroblast cells exhibited a spherical and non-flattened morphology, indicating the lack of cell adhesion to the biomaterial surface. The results suggest that the fabricated hydrogels were nontoxic and hindered cell growth on their surfaces. The described biological property, namely the lack of surface-promoting cell adhesion, is one of the main advantages of temporary external dressings that are frequently changed, as they will not lead to tissue damage at the wound site during replacement. Although this phenomenon may be surprising due to the widely known adhesive properties of polycatecholamines, this is in agreement with our previous results [24]. Our observations suggested that the adhesion of cells to polycatecholamine-modified hydrogels may vary depending on the modification method—adhesion is promoted when polycatecholamine is deposited onto the hydrogel surface and inhibited when the polycatecholamine network is distributed within the hydrogel, as in the case of this and our previous study [24]. Thus, it should be emphasized that the selection of an appropriate modification method may significantly affect the properties of the modified material.

**Figure 6:**
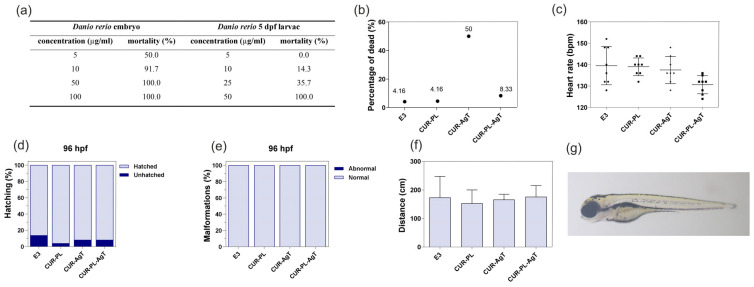
(**a**) Mortality of *Danio rerio* embryos and 5 dpf larvae in a concentration-dependent manner during silver nanoparticle exposure for 24 h (*n* = 24). Effect of CUR-PL, CUR-AgT, and CUR-PL-AgT exposure observed in *Danio rerio*: (**b**) the percentage of mortality (*n* = 24); (**c**) heart rate (beats per minute (BPM) (*n* = 10); (**d**) hatching rate (*n* = 12–20); (**e**) percentage of morphological alterations in 96 hpf larva (*n* = 12–20); (**f**) distance covered by *Danio rerio* larvae during locomotor activity assay; and (**g**) healthy *Danio rerio* organism without malformations. Data are presented as means ± SD; two-way ANOVA.

Further hydrogel biocompatibility was evaluated using a *Danio rerio* (zebrafish) in vivo model. The effect of silver nanoparticles (with a concentration range of 5–100 µg/mL) on zebrafish embryos was studied, and showed high mortality during the first 24 h of incubation (Figure 6a). Even the lowest concentration of nanoparticles (5 µg/mL) resulted in 50% mortality. However, the potential application of formulated hydrogel concerns the mature skin cells, which are less susceptible to different agents than fish embryos. Thus, older stages of zebrafish life (5 dpf) and a lower range of nanoparticles concentrations (5–50 µg/mL) were chosen for further studies. It was shown that the mortality rate in older larvae was lower than in the embryo stage, indicating that the 5 dpf zebrafish are more resilient to the effects of nanoparticles (Figure 6a).

Despite the significant toxicity of self-standing nanoparticles, biomaterials containing these structures were likely to exhibit less harmful effects due to their interactions. Therefore, the toxicological effect caused by exposure to NP-loaded hydrogels was evaluated using the modified Fish Embryo Acute Toxicity (FET) Test (OECD, 2013). The addition of AgT nanoparticles to the hydrogel structure (in CUR-AgT) elicited a mortality of 50.0% of the larvae (Figure 6b). However, CUR-PL-AgT exerted a lower mortality (8.33%) than CUR-AgT. This behavior suggested that polycatecholamine increases the safety of NP-loaded hydrogels. In the case of CUR-PL, the mortality was comparable to the E3 solution (both 4.16%), indicating that PL was nontoxic and mitigated the toxic effects of AgT nanoparticles. Among the surviving embryo population, some were strong enough to hatch, while others remained in their eggs.

The hatching rate for all modified variants was higher than for E3 (the control for this test), with the highest value observed for the CUR-PL hydrogel, suggesting its higher safety for zebrafish embryos (Figure 6d). The analysis showed a statistically significant influence on heart rate between E3 and CUR-PL-AgT (Figure 6c) (two-way ANOVA results, Table S2). Malformations were not observed in any of the groups (Figure 6e). A locomotor activity test was conducted to assess the impact of AgNP-loaded hydrogels on the central nervous system (Figure 6f). Figure 6g presents a healthy *Danio rerio* organism without malformations. The table presenting the distance covered by the experimental groups of organisms is included in the Supplementary Materials (Table S4). None of the materials affected the locomotor activity of the larvae, although CUR-PL induced a slight decrease in the observed parameter, which was not statistically significant (Table S3).

### 2.6. Hemocompatibility

The clot-forming ability of biomaterials is a crucial indicator of blood compatibility in terms of wound dressing biosafety, particularly in the context of contact with bleeding wounds. Therefore, the influence of the designed hydrogels on the clot-forming process was evaluated.

**Figure 7:**
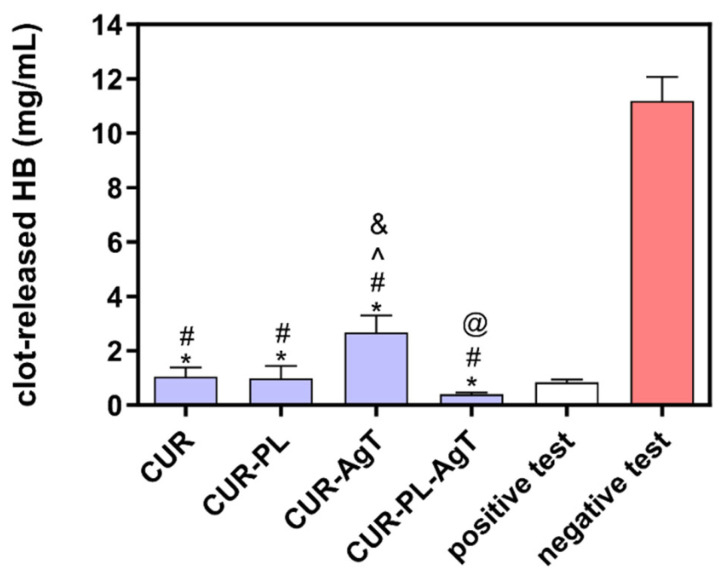
Clot formation in blood incubated with tested biomaterials after 30 min of contact. (*) indicates statistically significant differences between a positive test and the samples; (#) indicates statistically significant differences between a negative test and the samples, (^) indicates statistically significant results between CUR and the samples, (&) indicates statistically significant results between CUR–PL and the samples, and (@) indicates statistically significant results between CUR–AgT and the samples, according to one-way ANOVA with a post hoc Tukey’s test (*p* < 0.05).

Concerning clot formation (Figure 7) after 30 min., the clotting efficiency of all materials nearly matched the positive control, with CUR-AgT being the exception. However, the level of hemoglobin released from blood incubated with CUR-PL-AgT was the lowest compared to that of the other hydrogels, indicating the highest efficacy in clot formation. Overall, CUR-PL-AgT appears to be the most promising in terms of the design of potential hemostatic wound dressings.

### 2.7. Antibacterial Properties

A comprehensive analysis of the antibacterial properties of biomaterials designed for wound dressings is essential due to the risk of wound infection. Bacterial adhesion to materials is the initial step leading to biofilm formation, which can result in massive infection and the inflammatory complications of wounds [53,54]. The primary antibacterial mechanism of AgNPs is attributed to the fact that they can be ionized to Ag^+^, and both the AgNPs and the released Ag^+^ ions can contribute to the killing of bacterial cells [55,56,57]. Considering this mechanism, the antibacterial activity of silver-modified hydrogels is expected to be high.

**Figure 8:**
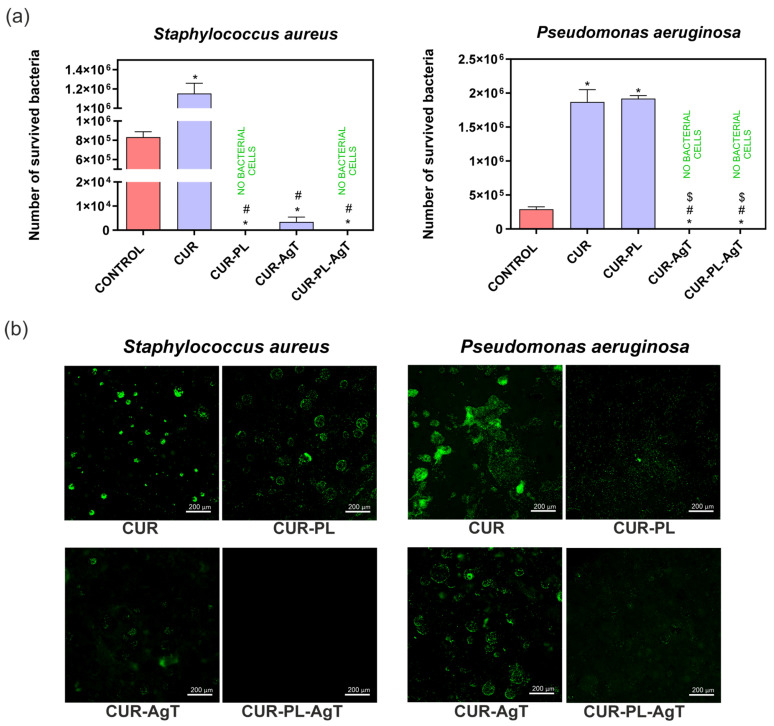
(**a**) Viability of bacteria in test according to the AATCC 100-2004 standard for porous materials, where (*) indicates statistically significant differences between the samples and the control for this test; (#) indicates statistically significant results between CUR and the samples; ($) indicates statistically significant results between CUR-PL and the samples, according to one-way ANOVA with post hoc Tukey’s test (*p* < 0.05); and (**b**) CLSM images of hydrogels after bacterial adhesion test (2 h).

According to the AATCC 100-2004 standard, the antibacterial activity showed interesting data (Figure 8a). The addition of poly(L-DOPA) to the matrices caused the complete death of *S. aureus* (both for CUR-PL and CUR-PL-AgT), but not *P. aeruginosa*. Interestingly, CUR-AgT showed lower antibacterial activity in a test performed according to the AATCC 100-2004 standard for porous materials against *S. aureus* than CUR-PL, suggesting that this strain is more susceptible to the polycatecholamine effect than AgTNPs. This antibacterial effect was expected because the antibacterial activity of polycatecholamines had already been reported [58,59]. In turn, *P. aeruginosa* does not seem sensitive to CUR-PL alone but is susceptible to CUR-AgT. However, the CUR-PL-AgT hydrogels killed the cells of both bacterial strains, suggesting the enhanced antibacterial activity of PL and AgT in combination.

Figure 8b shows that the control hydrogel (CUR) promoted bacterial adhesion of two strains common in wound infection: *Staphylococcus aureus* and *Pseudomonas aeruginosa*. However, curdlan modified with PL (CUR-PL) significantly reduced bacterial adhesion, particularly for *P. aeruginosa* (Figure 8b). The addition of AgT to the curdlan matrix (CUR-AgT) notably decreased the adhesion of *S. aureus*. When AgT was added to the poly(L-DOPA)-modified curdlan hydrogel (CUR-PL-AgT), the adhesion of *S. aureus* was inhibited entirely. At the same time, for *P. aeruginosa,* it was significantly reduced.

It seems surprising that CUR-PL significantly reduces *P. aeruginosa* adhesion without affecting the viability of this strain. However, this may be explained by the specific properties of both CUR-PL and the *P. aeruginosa* cell surface. PL is composed of L-DOPA monomers, which are rich in negatively charged carboxyl groups. On the other hand, Gram-negative bacteria (such as the *P. aeruginosa* strain) generally exhibit a more significant negative surface charge than Gram-positive bacteria, due to the presence of negatively charged lipopolysaccharides in their outer membrane (Gram-positive bacteria lack lipopolysaccharides in their cell wall) [60]. Hence, the repulsion between the negatively charged *P. aeruginosa* cells and hypothetically negatively charged PL could weaken the interactions between CUR-PL and *P. aeruginosa*, resulting in both limited antibacterial activity of the hydrogel and lowered bacterial adhesion to its surface.

To summarize, the CUR-PL-AgT hydrogel exhibited the strongest antibacterial effect, particularly in terms of adhesion and bacterial killing, likely due to the cumulative activity of polycatecholamine and AgTNPs.

## 3. Materials and Methods

### 3.1. Materials

Curdlan (isolated from cultures of *Alcaligenes faecalis*; cat. No. 281–80,531; DP 6790; Mw approx. 1100 kDa; specific rotation [A]^20^/_D_: +30 to +35; content of Cl^−^ < 0.5%, content of heavy metals < 0.002%) was supplied by Wako Chemicals (Osaka, Japan). TRIS (2-amino-2-(hydroxymethyl)propane-1,3-diol) and L-DOPA (3,4-dihydroxy-l-phenylalanine) were purchased from Sigma-Aldrich (St. Louis, MO, USA). Other reagents (of analytical grade) were supplied by Avantor (Gliwice, Poland), unless stated otherwise.

### 3.2. Methods

#### 3.2.1. Synthesis of the Nanoprism Ag Nanoparticles

Silver triangular nanoparticles (AgTNPs) were prepared using chemical reduction synthesis. The reaction mixture consisted of 100 μL of 0.1 M AgNO_3_ (99%, POCH, Poland), 1.5 mL of 0.1 M Na_3_C_6_H_5_O_7_ (trisodium citrate, TSC; >99%, Chempur, Piekary Śląskie, Poland), 280 μL of 30% H_2_O_2_ (POCH, Gliwice, Poland), and 100 mL of deionized water, and was stirred vigorously (500 rpm) for 10 min at room temperature. Subsequently, the stirring speed was reduced to 200 rpm, and 1 mL of a 0.1 M NaBH_4_ (99% Thermo Scientific, Warsaw, Poland) reducing agent was added, followed by an additional 10 min of mixing. The resulting silver nanoparticles were separated using a laboratory centrifuge and washed with fresh deionized water. The concentration of nanoparticles in the dispersion was determined using the microbalance method.

#### 3.2.2. Characterization of Ag Nanoparticles

The size and morphology of the nanoparticles were analyzed using a Tecnai Osiris X-FEG HRTEM microscope (FEI, Eindhoven, The Netherlands) operated at 200 kV. Sample preparation involved the deposition of a droplet of AgNPs containing a water suspension (0.25 mg/mL) on a 200-mesh copper grid coated with a thin carbon film (EM Resolutions, Newcastle, UK). Afterwards, a sample was air-dried overnight under dust protection. The size and their distribution were estimated using ImageJ 1.54j software. The absorption spectrum of the AgTNPs was measured with an Ocean Optics FLAME-S-VIS-NIR spectrophotometer (Ocean Optics, New York, NY, USA) in the range of 500–950 nm using a quartz cuvette with a 10 mm optical path.

#### 3.2.3. Synthesis of Hydrogel Samples

**Figure 9:**
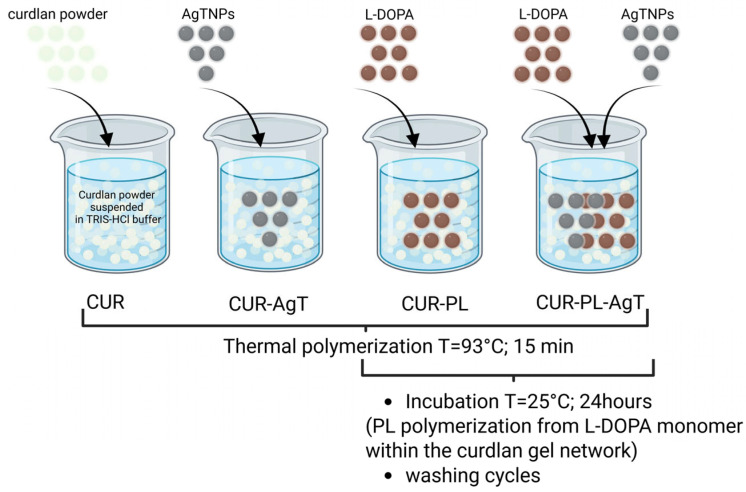
Schematic representation of samples synthesis. This figure was created using BioRender.com.

Hydrogels were synthesized according to the procedure described elsewhere [32] with modifications depending on the sample type (Figure 9). Briefly, for control hydrogel (CUR), curdlan powder was suspended in Tris/HCl buffer (pH 8.5). In the case of PL-modified hydrogel (CUR-PL), curdlan powder was suspended in Tris/HCl buffer (pH 8.5) with the subsequent addition of L-DOPA monomer (2 mg/mL) and stirred until L-DOPA was completely dissolved. The NP-enriched hydrogel (CUR-AgT) was prepared by curdlan powder suspended in Tris/HCl buffer (pH 8.5) with the addition of the AgTNP solution (final concentration of 3 mg/g dry mass). The NP-enriched and PL-modified hydrogel (CUR-PL-AgT) was obtained by combining the steps for CUR-PL, with the NP solution (at a final concentration of 3 mg/g dry mass) added 15 min after L-DOPA introduction. All final suspensions were polymerized at 93 °C for 15 min and cooled to perform the basic synthesis step—the thermal setting of the curdlan polymer network. After cooling, all hydrogels were cut into 3 mm slices. In case of CUR-PL and CUR-PL-AgT hydrogels, the slices were further incubated at 25 °C for 24 h in air to perform the second synthesis step—the PL polymerization from an L-DOPA monomer within the curdlan gel network.

Then, the slices of all hydrogels were washed 10 times in 100 mL DI H_2_O, frozen, and lyophilized (SRK, System Technik GMBK, Riedstadt, Germany). Before cell cultures and the zebrafish and antibacterial activity experiments, all hydrogels were sterilized by the ethylene oxide method in a paper/plastic peel pouch (sterilization for 1 h at 55 °C; aeration for 20 h). Photos of all the prepared hydrogels can be found in the Supplementary File (after mechanical tests).

#### 3.2.4. NIR Energy Conversion on Hydrogels

The light-to-heat conversion on composites was performed using a setup equipped with an 808 nm continuous laser source. NIR radiation was delivered through 400 μm optical fiber (CNI, Changchun, China), while thermal effects were recorded using a FLIR T660 thermovision camera (FLIR, Wilsonville, OR, USA). The measurements were carried out inside an insulated polystyrene box to minimize heat exchange with the environment. The laser power was calibrated with an Ophir StarLite power meter with a beam track thermal sensor 10 A-PPS (Ophir, Jerusalem, Israel). For all measurements, a laser power within the range of 500–1400 mW and corresponding light optical densities of LOD 1–2.8 W/cm^2^ was used. Temperature evolution after stimulation with NIR light was recorded through a thermovision camera controlled by dedicated ResearchIR 4 software (FLIR, Wilsonville, OR, USA), while final data curation and analysis were performed with OriginPro2019 software (OriginLab Corporation, Northampton, MA, USA).

#### 3.2.5. Nanoparticles and Ions Release

Nanoparticle release from hydrogels (CUR-AgT and CUR-PL-AgT) was performed in a closed-loop system, as described elsewhere [32]. The concentration of nanoparticles was measured spectrophotometrically at 810 nm and quantified based on calibration curves. The cumulative release profile of the nanoparticles was calculated based on the results of 2 independent experiments (each in triplicate), based on the percentage of nanoparticles released from the samples at defined time points.

Ag^+^ ion release from the samples (CUR-AgT and CUR-PL-AgT) was tested in a semi-open-loop system using sterile PBS pH 7.4 (proportion: 5 mL PBS/0.1 g dry weight of hydrogel sample) at 37 °C, with agitation (50 rpm, Innova 42, New Brunswick Scientific, Edison, NJ, USA). Extracts collected from the hydrogels (15% of medium volume) were collected daily and replaced by the same volume of fresh PBS. The release test was carried out in triplicate, in 2 independent experiments. Then, the Ag^+^ ion concentration was measured in extracts collected daily until detectable, as described previously [32]. Briefly, the silver concentration was analyzed using the inductively coupled plasma optical emission spectrometry (ICP-OES) with the Varian 720-ES axial system (Varian Inc., Palo Alto, CA, USA). Calibration was performed using three standards (1, 2.5, and 5 mg/dm^3^) and a blank solution. The ICP-OES conditions: 1.0 kW power, plasma gas flow rate of 15.0 dm^3^/min, optical resolution of 0.004 nm, pump speed at 15 rpm, 10 s replicate read time, 18 s sample uptake delay, and three replicates. The analytical wavelength for silver was 328.068 nm with a detection limit of 0.005 mg/dm^3^.

#### 3.2.6. Mechanical Tests

Mechanical tests of all hydrogel samples were performed, as described elsewhere [32], using the universal testing machine EZ Test EX-SX (Shimadzu, Kyoto, Japan) equipped with the Trapezium program and a force sensor of 100 N. The compression test and stress relaxation test were carried out for hydrogel samples (ø = 13 mm, h = 15 mm; *n* = 10) completely soaked in PBS pH 7.4 with a 5 mm/min crosshead rate. Compressive strength (*σ_c_*_100N_) was measured as the maximum stress at 100 N. The relaxation test allowed for the determination of compressive strength (*σ_c_*_50%_), non-relaxed stress *σ_t_*_50%_, and non-relaxed relative stress *σ_w_*_50%_ calculated as the ratio *σ_t_*_50%_/*σ_c_*_50%_.

#### 3.2.7. Surface Properties

##### Wettability Characterization

A DSA 30 goniometer (Kruss GmbH, Hamburg, Germany) was used to characterize wettability. In the test, a static contact angle method was applied using ultrapure water (sessile drop technique) obtained from the Milli-Q^®^ Water Purification System (Merck, Warsaw, Poland). The wetting behavior measurements were carried out for at least four independent samples.

##### Surface Roughness Parameters

The morphology of biomaterials and surface area roughness were visualized and measured using an Olympus LEXT OLS5100 3D laser scanning microscope (Olympus Corporation, Tokyo, Japan). Seven independent measurements were made for each sample. The biomaterial surfaces were observed at 211× magnification in ten different areas. The tested areas’ dimensions were 1281 × 1280 μm, and samples were scanned using a LEXT OLS5100 3D confocal laser scanning microscope.

##### Characterization of the Porosity

X-ray computed microtomography (μ-CT, Skyscan 1272 version 1.5 microtomography, Bruker, Billerica, MA, USA) measurements were carried out at a 50 kV voltage and a 200 µA current without a filter, and the projected images were collected every 0.5° over 360° with an exposure time of 735 ms, obtaining an isotropic voxel size (voxel resolution) of 2.5 µm. The NRecon 1.7.0.4 software (Bruker, USA) was used to reconstruct the samples by alignment adjustment, beam hardening correction, and ring artifacts filtering. The porosity analysis was carried out using CTAn 1.20.8.0 software (Bruker, USA). Specifically, the image was binary segmented, smoothed by eliminating white/black speckles of less than 15 voxels, and finally, the porosity was calculated. Parallel to that, the CTvox 3.2 software was used to render 3D reconstructions (Bruker, USA).

#### 3.2.8. Cytotoxicity Evaluation

An experiment was carried out using a BJ cell line (normal human skin fibroblasts) obtained from ATCC (American Type Culture Collection, Teddington, UK). The cells were cultured in EMEM medium (ATCC-LGC Standards, Teddington, UK) supplemented with 10% FBS (Pan-Biotech GmbH, Aidenbach, Bavaria, Germany), 100 U/mL penicillin, 100 μg/mL streptomycin (Sigma-Aldrich Chemicals, Warsaw, Poland), and maintained at 37 °C in a humidified atmosphere of 5% CO_2_ and 95% air.

The cytotoxicity of the produced biomaterials was tested indirectly according to ISO 10993-5:2009 using fluid extracts obtained in the short-term experiment. The high absorption capacity and the swelling tendency of the produced samples resulted in the reduction in the ratio between the sample weight and the volume of the extraction vehicle compared to the mentioned ISO standard. A 60 mg/mL proportion was used in the experiment. The BJ cells were seeded in 96-multiwell plates in 100 μL of the complete culture medium at a concentration of 2 × 10^4^ cells per well and cultured for 24 h. The culture medium was then replaced with the extracts prepared from the produced biomaterials. Polypropylene extract was prepared and served as a negative control for cytotoxicity. After 24 h of incubation, the growth medium was replaced with 100 μL of appropriate extracts. The cells were maintained with extracts for 24 h, at which point BJ viability was evaluated using the MTT (Sigma-Aldrich Chemicals, Warsaw, Poland) and total LDH (Sigma-Aldrich Chemicals, Warsaw, Poland) tests. The MTT test was conducted according to the procedure described earlier [61]. The total LDH test was performed according to the manufacturer’s instructions using an LDH cytotoxicity kit. The results of both tests were presented as the percentage of negative control of cytotoxicity. The indirect cytotoxicity tests were repeated in three independent experiments.

Biomaterials cytotoxicity was also estimated in a direct contact method test using a Live/Dead Double Staining Kit (Sigma-Aldrich Chemicals, St. Louis, MO, USA). For this purpose, biomaterials were sterilized by ethylene oxide and placed in the wells of the 48-multiwell plate. Then samples were presoaked using a complete culture medium with 500 μL of EMEM medium containing 1 × 10^5^ BJ fibroblasts seeded directly onto the biomaterials. Cells cultured on a polystyrene plate served as a control. After 48 h of culture at 37 °C, cells were stained using the Live/Dead Double Staining Kit in accordance with the manufacturer’s procedure. The Calcein-AM and propidium iodide (PI) dyes were used in the test. After staining, viability was estimated based on confocal laser scanning microscope analysis (CLSM, Olympus Fluoview equipped with FV1000, Olympus Corporation, Tokyo, Japan).

#### 3.2.9. In Vivo Experiments—*Danio rerio* Model

*Danio rerio* of the AB strain were maintained at the Experimental Medicine Center, Medical University of Lublin, Poland, at 28.5 °C, on a 14/10 h light–dark cycle under standard aquaculture conditions. Fertilized eggs were collected by natural spawning. Embryos were reared in embryo medium E3 (pH 7.1–7.3; 17.4 µM NaCl, 0.21 µM KCl, 0.12 µM MgSO_4_, and 0.18 µM Ca(NO_3_)_2_) in an incubator at 28.5 °C. Immediately after the experiment, larvae were killed by immersion in 15 μM tricaine solution. All details were described by Michalicha et al. 2021 [24]. All experiments were carried out in accordance with the National Institute of Health Guidelines for the Care and Use of Laboratory Animals and the European Community Council Directive for the Care and Use of Laboratory Animals of 22 September 2010 (2010/63/EU). Experiments with larvae older than 120 h post fertilization (hpf) were approved by the Local Ethics Committee in Lublin, Poland (Permission No: 44/2022; 14 March 2022). For the experiment with larvae up to 120 hpf, the agreement of the Local Ethical Commission was not required.

The zebrafish embryo toxicity assay followed the modified OECD Guidelines for Chemical Testing (OECD, 2013). The toxicity of ethanol, used as a solvent, was evaluated in our previous work [32] and revealed that the alcohol concentration used in the present studies did not induce mortality. Embryos not older than 90 min post fertilization were examined under a light microscope (Stemi 508, Zeiss, Oberkochen, Germany), and viable fertilized embryos were carefully selected and then transferred to 96-well plates within 3 hpf. Each embryo was individually incubated in 200 µL of the compounds or control solutions. The embryos were exposed to either ‘control’ or ‘treatment’ solutions for 96 h. Throughout this period, the embryos were observed every 24 h using a stereomicroscope, and the following parameters were recorded: survival rate, hatching rate, and the presence of any developmental abnormalities. At 96 hpf, the following measurements were taken: the number of deceased embryos, the presence of malformations, the hatching rate, and the heart rate. To measure heart rate, the larvae were allowed to equilibrate at room temperature for 30 min, and then their heartbeats were counted under a stereomicroscope for 15 s. The values obtained were multiplied by four to determine the heart rate in beats per minute (bpm).

Evaluation of locomotor activity was performed in 5 days post-fertilization (dpf) larvae, after the FET test, with one larva in each well of a 96-multiwell plate. EthoVision XT 17 video tracking software (Noldus, EthoVision XT 17) was used for evaluating locomotor activity. The distance moved in 10 min period was calculated in a light condition.

Obtained data were statistically analyzed using one-way and two-way analysis of variance followed by Tukey’s post hoc test or Bonferroni’s post hoc test, respectively. The confidence limit of *p* < 0.05 was considered statistically significant. The analysis was carried out using GraphPad Prism v8.3.1 software.

#### 3.2.10. Clot Formation Test

For the clot formation test, human citrated blood was obtained from a healthy volunteer (procedure approved by the Bioethics Committee at the Medical University of Lublin, no. KE-0254/258/2020; 26 November 2020). Its total hemoglobin and plasma hemoglobin concentration (measured by the reaction with Drabkin reagent and appropriate calibration curve), using 96-well plates, and Synergy H4 hybrid microplate reader (Biotek, Winooski, VT, USA) were 1.38 mg/mL and 0.13 mg/mL, respectively. As a negative control for the test, non-activated Ca^2+^-free whole blood was used. The positive control for the test was 30 mg ± 2 mg of HDPE pieces.

The test was carried out on 30 mg ± 2 mg samples of all hydrogels (each variant in triplicate), as described elsewhere [24].

#### 3.2.11. Antibacterial Activity Evaluation

##### Bacterial Strains and Maintenance

*Staphylococcus aureus ATCC 25923* and *Pseudomonas aeruginosa ATCC 27853* reference bacterial strains were grown at 37 °C for 24 h, in Mueller–Hinton Agar medium (Biomaxima, Poland). Then, the bacteria were suspended in a sterile 0.9% NaCl or Mueller–Hinton (M-H) broth (Biomaxima, Lublin, Poland) at the desired density.

##### Antibacterial Activity Test (Based on the Standard: AATCC Test Method 100-2004)

The antibacterial activity assessment followed a protocol described by Michalicha et al. [24]. The hydrogels were exposed to bacterial suspensions (1.5 × 10^5^ CFU/mL) of each strain, in quadruplicate, in volumes adjusted to absorption capacity of each hydrogel. After incubation (37 °C, 24 h), the hydrogel samples were transferred to sterile 0.9% NaCl and subjected to vigorous shaking (1 min) to elute bacterial cells from the hydrogel network. As a control, the equivalent volume of bacterial inoculum was incubated. The collected eluates were then plated onto M-H agar in triplicate using an automatic plater (EasySpiral Dilute, Interscience, Saint-Nom-la-Bretéche, France). After incubation (37 °C, 48 h), the colony-forming units (CFUs) were enumerated using a Scan 300 counter.

##### Bacterial Adhesion Test

The test was carried out according to the protocol described earlier [24,62].

## 4. Conclusions

Our results indicate that the applied modification of a curdlan hydrogel with the addition of shape-defined AgNPs and poly(L-DOPA) is a promising starting point for producing biosafe and antibacterial wound dressing materials with improved mechanical performance. The heat generation ability was reinforced by adding L-DOPA to the hydrogel structure. All hydrogel matrices were found to be non-toxic toward eukaryotic cells. Regarding biosafety in the zebrafish larvae model, adding PL to AgTNP-modified hydrogels reduced the larval mortality caused by the nanoparticles. The hydrogel with both PL and AgTNPs (CUR-PL-AgT) demonstrated its superiority over the hydrogel with AgTNPs alone in terms of blood clotting and overall antibacterial properties. Taking into account all the observed biological effects and the fact that the hydrogels counteracted fibroblast adhesion, it seems logical that the CUR-PL-AgTNP hydrogel may be an interesting proposition for the design of hydrogel dressings for infected and bleeding wounds.

## Data Availability

Data will be made available on request.

## Supplementary Materials

The following supporting information can be downloaded at: https://www.mdpi.com/1420-3049/31/1/181/s1. Table S1. Pore size distribution. Table S2. Two-way ANOVA statistical analysis of CUR-PL, CUR-Agt, and CUR-PL-AgT exposure observed in *Danio rerio* 5 dpf (days post fertilization) larvae (*n* = 10) on heart rate. Table S3. Two-way ANOVA statistical analysis of CUR-PL, CUR-AgT, and CUR-PL-AgT exposure observed in *Danio rerio* 5 dpf (days post fertilization) larvae (*n* = 24) on locomotor activity [cm/10 min]; Data are presented as means ± SD. Table S4. Effect of CUR-PL, CUR-Agt, and CUR-PL-AgT exposure observed in *Danio rerio* 5 dpf (days post fertilization) larvae (*n* = 24) on locomotor activity [cm/10 min]. Data are presented as means ± SD. Figure S1. Photograph of all the hydrogel samples (after mechanical testing).
