# Supplementary material for: Poly(levodopa)-Modified β-(1 → 3)-D-Glucan Hydrogel Enriched with Triangle-Shaped Nanoparticles as a Biosafe Matrix with Enhanced Antibacterial Potential
Source: Molecules. 2026 Jan 3;31(1):181. doi: 10.3390/molecules31010181 (PMC13319943; doi:10.3390/molecules31010181)
Supplement: Supplementary file 1 [file molecules-31-00181-s001.zip › molecules-4021781-supplementary.pdf]

Supplementary file

Poly(levodopa)-modified β-(1→ 3)-D-glucan hydrogel enriched with triangle-shaped nanoparticles as a biosafe matrix with enhanced antibacterial potential

Anna Michalicha[1*], Vladyslav Vivcharenko[2], Anna Tomaszewska[3], Magdalena Kulpa-Greszta[3], Barbara Budzyńska[4], Dominika Fila[5], Judit Buxadera-Palomero[6,7], Agnieszka Krawczyńska[9], Cristina Canal[6,7,8], Dorota Kołodyńska[5], , Anna Belcarz-Romaniuk[1], Robert Pązik[3*]

[1]Chair and Department of Biochemistry and Biotechnology, Medical University of Lublin, Chodzki 1, 20-093 Lublin, Poland; anna.michalicha@umlub.edu.pl; anna.belcarz-romaniuk@umlub.edu.pl

[2]Department of Tissue Engineering and Regenerative Medicine, Medical University of Lublin, Chodzki 1, 20-093 Lublin, Poland; vladyslav.vivcharenko@umlub.edu.pl

[3]Faculty of Biotechnology, Collegium Medicum, University of Rzeszow, Pigonia 1, 35-310 Rzeszow, Poland;

atomaszewska@ur.edu.pl; mkulpa@ur.edu.pl; rpazik@ur.edu.pl

[4]Independent Laboratory of Behavioral Studies, Medical University of Lublin, Chodzki 1, 20-093 Lublin, Poland; barbarabudzynska@umlub.pl

[5]Department of Inorganic Chemistry, Institute of Chemical Sciences, Faculty of Chemistry, Maria Curie-Skłodowska University, Maria Curie-Skłodowska Sq. 2, 20-031 Lublin, Poland; dominika.fila@mail.umcs.pl; dorota.kolodynska@mail.umcs.pl

[6]Biomaterials, Biomechanics and Tissue Engineering Group, Department of Materials Science and Engineering and Institute for Research and Innovation in Health (IRIS), Universitat Politècnica de Catalunya BarcelonaTech (UPC), Av. Eduard Maristany 10-14, 08019 Barcelona, Spain; judit.buxadera@upc.edu; cristina.canal@upc.edu

[7]Barcelona Research Center in Multiscale Science and Engineering (CCEM), UPC, Barcelona, Spain.

[8]Centro de Investigación Biomédica en Red de Bioingeniería, Biomateriales y Nanomedicina (CIBER-BBN), Instituto de Salud Carlos III, Spain.

[9]Faculty of Materials Science and Engineering, Warsaw University of Technology, 02-507 Warsaw, Poland agnieszka.krawczynska@pw.edu.pl

Table S1 Pore size distribution

	**% porosity in range**			
**Mid-range (um)**	CUR	CUR-PL	CUR-AgT	CUR-PL-AgT
**5**	0.3794	0.8655	0.162	0.1757
**10**	1.6022	2.3687	0.8356	0.7869

**15**	2.2007	2.6621	1.3993	1.2804
**20**	3.7806	5.1044	3.3322	2.882
**25**	4.5252	6.2438	5.1181	4.2227
**30**	4.7433	6.2123	6.3482	5.0311
**35**	6.0979	7.7081	9.6741	7.5516
**40**	6.326	7.6248	11.0865	8.5311
**45**	6.14	7.2056	11.3029	8.7975
**50**	6.2088	7.0729	11.3041	9.21
**55**	6.4194	6.8546	10.4316	9.2485
**60**	5.8101	5.856	8.1259	7.6845
**65**	5.4097	5.4807	6.6701	7.2443
**70**	4.7817	4.7844	4.8526	6.1236
**75**	4.5046	4.0149	3.2186	4.3116
**80**	3.8378	3.4051	2.2379	3.7815
**85**	3.9977	2.8732	1.5545	2.8681
**90**	3.1627	2.3804	0.9572	2.1828
**95**	3.1789	2.0439	0.5592	1.7425
**100**	2.3507	1.6609	0.3507	1.3141
**105**	2.0241	1.3753	0.2008	1.0891
**110**	1.5929	1.0079	0.1323	0.8012
**115**	1.6575	0.8229	0.0466	0.6235
**120**	1.1303	0.8371	0.0471	0.4832
**125**	1.214	0.6171	0.0232	0.3144
**130**	0.8008	0.4278	0.0213	0.4234
**135**	0.8001	0.3837	0.0074	0.3785
**140**	1.023	0.3344		0.2068
**145**	0.8928	0.4387		0.1528
**150**	0.4218	0.2381		0.1237
**155**	0.3283	0.2665		0.1784
**160**	0.3501	0.1495		0.0714
**165**	0.3939	0.1504		0.0799
**170**	0.1808	0.1025		0.0403
**175**	0.1884	0.078		0.0628
**180**	0.0441	0.0776		
**185**	0.2064	0.0331		
**190**	0.045	0.0306		
**195**	0.0472	0.0172		
**200**	0.2051	0.0171		
**205**	0.0673	0.1075		
**210**	0.2928	0.0032		
**215**	0.3282	0.0615		
**220**	0.0312			
**225**	0.2765			

Table S2 Two-way ANOVA statistical analysis of CUR-PL, CUR-Agt, and CUR-PL-AgT exposure observed in Danio rerio 5 dpf (days post fertilization) larvae (n=10) on heart rate.

two-way ANOVA	F (d,f)	p
Interactions	(1, 28) = 2,088	=0,1595
CUR-PL	(1, 28) = 5,531	=0,0260
CUR-AgT	(1, 28) = 2,795	=0,1057

Table S3 Two-way ANOVA statistical analysis of CUR-PL, CUR-AgT, and CUR-PL-AgT exposure observed in Danio rerio 5 dpf (days post fertilization) larvae (n=24) on locomotor activity [cm/10 min]; Data are presented as means ± SD.

two-way ANOVA	F (d,f)	p
Interactions	(1.77)=1.65	=0.20
CUR-PL	(1.77)=0.19	=0.66
CUR-AgT	(1.77)=0.43	=0.51

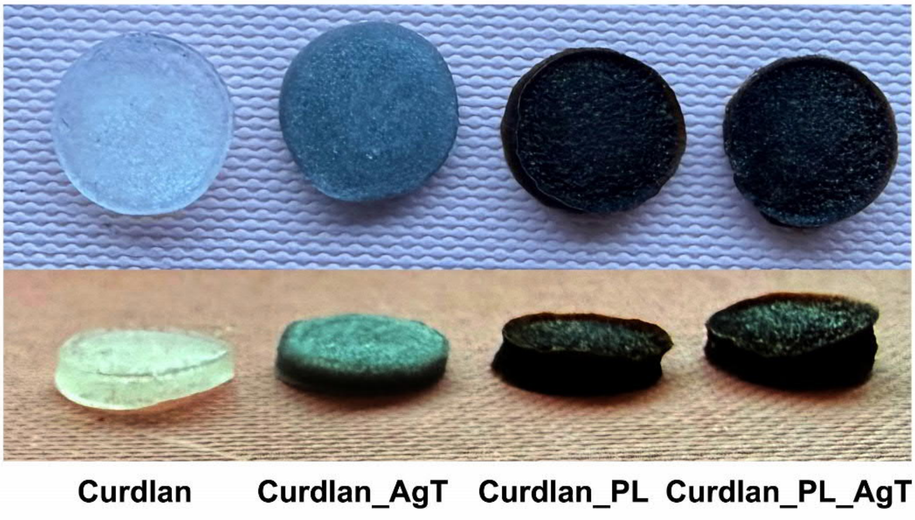

Figure 1s. Photograph of all the hydrogel samples (after mechanical testing)

Table S4 Effect of CUR-PL, CUR-Agt, and CUR-PL-AgT exposure observed in Danio rerio 5 dpf (days post fertilization) larvae (n=24) on locomotor activity [cm/10 min]. Data are presented as means ± SD.

	**E3**	**CUR-PL**	**CUR-AgT**	**CUR-PL-AgT**
**Distance [cm/10min]**	173.9±15.1	153.4±9.8	166.4±5.2	176.4±8.5
